# Supplementary material for: “Maybe it’s Not Just the Food?” A Food and Mood Focus Group Study
Source: Int J Environ Res Public Health. 2023 Jan 21;20(3):2011. doi: 10.3390/ijerph20032011 (PMC9915006; doi:10.3390/ijerph20032011)
Supplement: Supplementary file 1 [file ijerph-20-02011-s001.zip › ijerph-2170832-supplementary.pdf]

## COREQ (COnsolidated criteria for REporting Qualitative research) Checklist

A checklist of items that should be included in reports of qualitative research. You must report the page number in your manuscript where you consider each of the items listed in this checklist. If you have not included this information, either revise your manuscript accordingly before submitting or note N/A.

| Topic | Item No. | Guide Questions/Description | Reported on Page No. |
|---|---|---|---|
| **Domain 1: Research team and reflexivity** | | | |
| *Personal characteristics* | | | |
| Interviewer/facilitator | 1 | Which author/s conducted the interview or focus group? | 6 |
| Credentials | 2 | What were the researcher's credentials? E.g. PhD, MD | 6 |
| Occupation | 3 | What was their occupation at the time of the study? | 6 |
| Gender | 4 | Was the researcher male or female? | 6 |
| Experience and training | 5 | What experience or training did the researcher have? | 6 |
| *Relationship with participants* | | | |
| Relationship established | 6 | Was a relationship established prior to study commencement? | 5 |
| Participant knowledge of the interviewer | 7 | What did the participants know about the researcher? e.g. personal goals, reasons for doing the research | 5 |
| Interviewer characteristics | 8 | What characteristics were reported about the inter viewer/facilitator? e.g. Bias, assumptions, reasons and interests in the research topic | 5 |
| **Domain 2: Study design** | | | |
| *Theoretical framework* | | | |
| Methodological orientation and Theory | 9 | What methodological orientation was stated to underpin the study? e.g. grounded theory, discourse analysis, ethnography, phenomenology, content analysis | 4 |
| *Participant selection* | | | |
| Sampling | 10 | How were participants selected? e.g. purposive, convenience, consecutive, snowball | 5 |
| Method of approach | 11 | How were participants approached? e.g. face-to-face, telephone, mail, email | 5 |
| Sample size | 12 | How many participants were in the study? | 5 |
| Non-participation | 13 | How many people refused to participate or dropped out? Reasons? | 5 |
| *Setting* | | | |
| Setting of data collection | 14 | Where was the data collected? e.g. home, clinic, workplace | 4 |
| Presence of nonparticipants | 15 | Was anyone else present besides the participants and researchers? | 6 |
| Description of sample | 16 | What are the important characteristics of the sample? e.g. demographic data, date | 5 |
| *Data collection* | | | |
| Interview guide | 17 | Were questions, prompts, guides provided by the authors? Was it pilot tested? | 6 |

| | | | |
|---|---|---|---|
| Repeat interviews | 18 | Were repeat inter views carried out? If yes, how many? | 6 |
| Audio/visual recording | 19 | Did the research use audio or visual recording to collect the data? | 6 |
| Field notes | 20 | Were field notes made during and/or after the inter view or focus group? | 6 |
| Duration | 21 | What was the duration of the inter views or focus group? | 6 |
| Data saturation | 22 | Was data saturation discussed? | 6 |
| Transcripts returned | 23 | Were transcripts returned to participants for comment and/or | 6 |
| **Topic** | **Item No.** | **Guide Questions/Description** | **Reported on Page No.** |
| | | correction? | |
| **Domain 3: analysis and findings** | | | |
| *Data analysis* | | | |
| Number of data coders | 24 | How many data coders coded the data? | 7 |
| Description of the coding tree | 25 | Did authors provide a description of the coding tree? | 7 |
| Derivation of themes | 26 | Were themes identified in advance or derived from the data? | 7 |
| Software | 27 | What software, if applicable, was used to manage the data? | 7 |
| Participant checking | 28 | Did participants provide feedback on the findings? | 6 |
| *Reporting* | | | |
| Quotations presented | 29 | Were participant quotations presented to illustrate the themes/findings? Was each quotation identified? e.g. participant number | 8-18 |
| Data and findings consistent | 30 | Was there consistency between the data presented and the findings? | 8-18 |
| Clarity of major themes | 31 | Were major themes clearly presented in the findings? | 8-18 |
| Clarity of minor themes | 32 | Is there a description of diverse cases or discussion of minor themes? | 18-23 |

Developed from: Tong A, Sainsbury P, Craig J. Consolidated criteria for reporting qualitative research (COREQ): a 32-item checklist for interviews and focus groups. *International Journal for Quality in Health Care*. 2007. Volume 19, Number 6: pp. 349 – 357

**Once you have completed this checklist, please save a copy and upload it as part of your submission. DO NOT include this checklist as part of the main manuscript document. It must be uploaded as a separate file.**

## Focus Group Questions

Thank you

Introduction – myself/research

Why this research is important

Ground rules – no wrong/right answer, don't talk over each other, what is said stays in this room/confidentiality

Explain about and ask permission for recording

**Opening Question**

- Can you tell the group why you decided to take part in this research

**Introductory Questions**

- How do you decide what is healthy and what is not? Where do we get this information?
- Prompt – Has anything changed over time in your understanding of what foods and drinks you consider healthy?
- Prompt - Has anything changed over time in your understanding of what foods and drinks you consider unhealthy?

I am now going to ask some questions that look at the relationship between food and mood

**Transition Questions**

- How do you think food and mood are related?

**Key Questions - Prompts**

- Can you describe how something you ate resulted in changing the way you felt?
- Can you describe how your feelings and emotions have influenced your eating?
- What would you say motivates you to eat healthily?
- What would you say makes you eat unhealthily?
- What aspects of your family or culture that are linked to the way you eat?

**Ending Question**

- What do you think the government could do to increase education about mood and food in Australia

**Table S1**

*Thematic Template Analysis*

| Notes | Codes | Preliminary Themes |
|---|---|---|
| Food security – financial and time constraints<br>Mood affecting food choices<br>Procrastination leads to unhealthy choices and lower mood<br>Decision fatigue<br>Meal prepping<br>Ultra-processed Western diet decreases mood<br>Taste habituation to hyper salty/hyper sweet in Western diet<br>Unhealthy foods increase mood short-term, detrimental long-term<br>Life stability helps healthy food choices<br>Life instability leads to unhealthy food choices | Time restrictions<br>Financial restrictions<br>Preparedness<br>Decision fatigue | Social Economics: Time, Finance and Food Security |
| Social context surrounding what, where, how and why you eat<br>Food habits learned from childhood<br>Parental role modelling<br>Eating alone decreases mood/nutrient absorption<br>Eating with loved ones increased mood<br>Foods healthier for mental health in a social context<br>Mediterranean culture – social aspect of food<br>Sociological construct – societal perceptions of food and mood<br>Geographical location – access to food<br>Traditional eating patterns – like your grandparents ate | Social Context<br>Upbringing<br>Culture | Social Context: Familial and cultural influences in food and mood |
| Preparing food with and for family increases mood<br>Memories of loved ones past<br>Food traditions passed through generations<br>Reminders of family<br>Comfort food<br>Food memories<br>Eating mindfully, rather than when distracted<br>Using childhood foods to self-medicate low mood<br>Foods used as reward or commiseration | Memories<br>Family<br>Mindful Eating<br>Nostalgia | Food Nostalgia: Unlocking Memories that Impact Mood |
